# Controllable alignment of elongated microorganisms in 3D microspace using electrofluidic devices manufactured by hybrid femtosecond laser microfabrication

**DOI:** 10.1038/micronano.2016.78

**Published:** 2017-02-27

**Authors:** Jian Xu, Hiroyuki Kawano, Weiwei Liu, Yasutaka Hanada, Peixiang Lu, Atsushi Miyawaki, Katsumi Midorikawa, Koji Sugioka

**Affiliations:** 1 RIKEN Center for Advanced Photonics, 2-1 Hirosawa, Wako, Saitama 351-0198, Japan; 2 Laboratory for Cell Function Dynamics, RIKEN Brain Science Institute, 2-1 Hirosawa, Wako, Saitama 351-0198, Japan; 3 Wuhan National Laboratory for Optoelectronics and School of Physics, Huazhong University of Science and Technology, Wuhan 430074, China; 4 Laboratory of Optical Information and Technology, School of Science, Wuhan Institute of Technology, Wuhan 430073, China

**Keywords:** dynamic imaging, electrofluidic devices, electro-orientation, femtosecond laser microfabrication, flagellar motions, selective metallization, 3D electric fields, 3D microfluidics

## Abstract

This paper presents a simple technique to fabricate new electrofluidic devices for the three-dimensional (3D) manipulation of microorganisms by hybrid subtractive and additive femtosecond (fs) laser microfabrication (fs laser-assisted wet etching of glass followed by water-assisted fs laser modification combined with electroless metal plating). The technique enables the formation of patterned metal electrodes in arbitrary regions in closed glass microfluidic channels, which can spatially and temporally control the direction of electric fields in 3D microfluidic environments. The fabricated electrofluidic devices were applied to nanoaquariums to demonstrate the 3D electro-orientation of *Euglena gracilis* (an elongated unicellular microorganism) in microfluidics with high controllability and reliability. In particular, swimming *Euglena* cells can be oriented along the *z*-direction (perpendicular to the device surface) using electrodes with square outlines formed at the top and bottom of the channel, which is quite useful for observing the motions of cells parallel to their swimming directions. Specifically, *z*-directional electric field control ensured efficient observation of manipulated cells on the front side (45 cells were captured in a minute in an imaging area of ~160×120 μm), resulting in a reduction of the average time required to capture the images of five *Euglena* cells swimming continuously along the *z*-direction by a factor of ~43 compared with the case of no electric field. In addition, the combination of the electrofluidic devices and dynamic imaging enabled observation of the flagella of *Euglena* cells, revealing that the swimming direction of each *Euglena* cell under the electric field application was determined by the initial body angle.

## Introduction

Dynamic observation and analysis of the motions of biological samples (cells, microorganisms, and so on) are important for investigating the functions of their specific body parts, such as the flagella of microorganisms, which help us to understand the locomotion mechanisms in many biological processes observed in the life sciences and to develop artificial microswimmers and bio-inspired systems^1–5^. One conventional method of performing such observations is to use an optical microscope combined with a high-speed camera^6–9^. For a detailed and in-depth analysis of the motions, observation from various angles is useful. Although observation along the direction perpendicular to the body motion (side view) has been carried out frequently, there have been few reports on observation along the parallel direction (front view), even though the latter provides a more functional analysis of flagellar movement. The introduction of microorganism suspensions into 3D microfluidic devices showed a potential for increasing the possibility of flexible observation owing to the spatial confinement of biological fluids^10^. However, in the case of microorganisms swimming along a specific direction in a continuous manner, observation efficiency is still not high enough, even when using microfluidic devices, because of the random swimming behavior of microorganisms. To maintain microorganism suspensions in good condition during observation experiments, the observation time of microorganism motions should be shortened to decrease the side effects of light illumination, whereas the acquisition rate of experimental data (images, videos, and so on) should be increased. Thus, to perform a flexible observation, including front-view observation, rapidly and with high efficiency, 3D nondestructive control of microorganism motion along a designable direction in microfluidic environments is highly desirable.

Electrical manipulation is an effective approach to controlling the motions of biological samples in microfluidic devices that offers many advantages, such as low cost, ease of operation, high controllability, and high efficiency. Thus, it enables a broad range of lab-on-a-chip applications in biological and medical research^11–16^. Among the many electromanipulation methods, electro-orientation offers a reproducible and nondestructive way of aligning elongated (asymmetric or non-spherical) cells/particles immersed in a solution of different permittivity using an electric field. Depending on the frequency of the applied electric field, dipole moments induced in the elongated cells determine their orientation/alignment either parallel or perpendicular to the direction of the electric field. The optimum frequencies for the orientation depend on the electrical and geometrical parameters of the cells and the conductivity of the cell suspensions. The electro-orientation of not only many elongated biological samples, such as *E. coli* (bacteria)^17,18^, *B. subtilis* (bacteria)^18^, *S. marcescens* (bacteria)^9^, *T. pyriformis* (ciliates)^19^, microtubules^20^, cardiac myocytes^21^, retinal photoreceptors^22^, ellipsoidal erythrocytes^23^, and yeast cells^24,25^, but also one-dimensional (1D) nanomaterials, such as nanowires and nanorods^26–28^, have been demonstrated. Electro-orientation in microfluidic chips has already been demonstrated for some important applications, such as the optical detection of bacteria^17^, the dielectric measurement of microtubules^20^, and cardiac tissue engineering^21^. The integration of electrodes into microfluidic chips is essential for the electro-orientation. Conventional electrode patterning techniques involving conductive film deposition combined with photolithography are capable of precisely preparing electrodes with controlled geometry and feature sizes; in most cases, however, the resulting electrodes are planar and situated in microchannels because of the inherently two-dimensional (2D) capability of these fabrication techniques. Flexible patterning of electrodes at any position in microfluidic channels provides more flexibility and extra functionality for electromanipulation in microfluidic environments^29^. Several approaches to fabricating and integrating electrodes with 3D configurations into microchannels have been attempted, primarily involving multiple lithographic steps combined with metal deposition and electroplating techniques^30,31^, metal ion implantation of a PDMS channel^32^, and in-channel injection of conductive liquid metals and composites^33–35^. The first two methods, however, require complex procedures involving the precise alignment and bonding of planar structures. Meanwhile, although the third method provides a simple way to integrate well-aligned electrodes, the mechanical stability and flexibility of electrodes prepared on the sidewalls of channels by this technique remain insufficient. Therefore, a simple and versatile technique for the flexible integration of electrodes into microfluidic chips is desirable for 3D electro-orientation and other electromanipulation methods.

We have previously developed hybrid subtractive and additive femtosecond (fs) laser microfabrication (fs laser-assisted wet etching (FLAE) of glass followed by water-assisted fs laser modification combined with electroless metal plating), which enables the selective metal deposition of microfluidic structures from the inside out without requiring any complicated photolithography, alignment, or bonding processes^36^. The resulting patterned metal structures feature strong adhesion to glass surfaces and high chemical stability. Furthermore, the introduction of water to the fs laser irradiation site (water-assisted fs laser modification) for the metallization process greatly improves the controllability and flexibility of the patterning process of electrodes prepared on the sidewalls in terms of their installed positions and geometries^37^. To the best of our knowledge, hybrid fs laser microfabrication based on nonlinear multiphoton absorption is the only technique that can perform 3D metal patterning in closed microchannels in a simple, flexible, reproducible, and bonding-free manner, although few applications of this technique have been demonstrated. Thus, it is important to demonstrate that this technique has the ability to manufacture new and practical electrofluidic devices, for instance, a device that can control electric fields in a 3D microspace of microfluidics for flexible manipulation of elongated cells. In this paper, we demonstrate the fabrication of new electrofluidic devices in which designable geometries of electrodes are integrated at the desired positions in glass microchannels by hybrid fs laser microfabrication. The fabricated electrofluidics are applied to nanoaquariums, which are a type of biochip for the dynamic observation and investigation of the function of aquatic microorganisms^10^. The flexible orientation of microorganisms in both the planar and *z*-directions (parallel and perpendicular to the device surface, respectively) is demonstrated, and the manipulation efficiency is quantitatively analyzed. The fabricated electrofluidic devices enable an efficient observation of the swimming of *z*-directionally oriented cells, significantly reducing the time needed to observe these cells from the front. In addition, observation using this device enables us to determine the position of flagella to elucidate the factor that determines the swimming direction of *Euglena* cells under an electric field.

## Materials and methods

### Monolithic fabrication of electrofluidic devices

**Figure 1:**
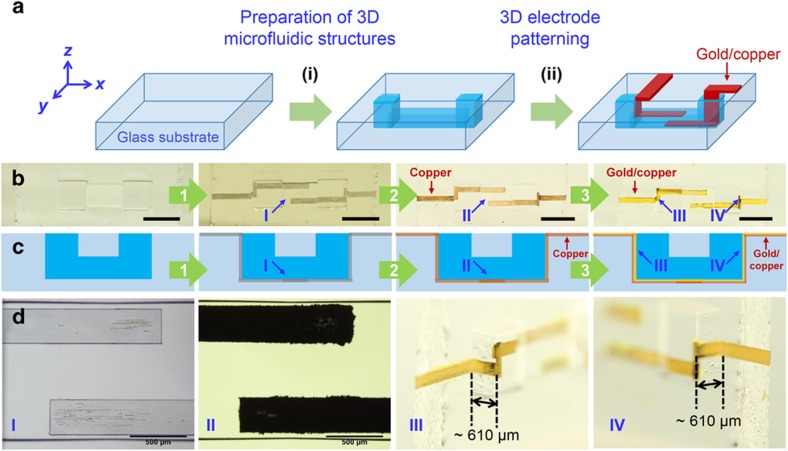
(**a**) Schematic of the fabrication procedure for electrofluidic devices using hybrid subtractive and additive fs laser microfabrication. (i) Preparation of 3D microfluidic structures in glass using fs laser-assisted wet chemical etching combined with post annealing for surface smoothing. (ii) 3D metal electrode patterning in fabricated glass microfluidic structures using water-assisted fs laser direct-write ablation followed by electroless metal plating. The red sections represent the patterned metals (gold/copper). (**b**) Photos of samples prepared at each step (top view of the chip structure), and (**c**) schematics of the corresponding steps (cross-sectional view of the chip structure). Process 1 represents water-assisted fs laser space-selective modification of a 3D glass microchannel. Processes 2 and 3 represent successive electroless copper and gold plating, respectively. The scale bar in each photo of (**b**) indicates 2 mm. (**d**) Close-up view (optical micrographs) of regions I and II and tilted view (photos) of electrodes patterned on the sidewalls of III and IV in (**b**).

As illustrated in Figure 1a, hybrid fs laser microfabrication of electrofluidic devices involves two main procedures of FLAE and water-assisted fs laser modification combined with electroless metal plating on glass substrates. The first procedure is the preparation of 3D microfluidic structures in glass. 10×10×2 mm photostructurable glass (Foturan®, Schott Glass Corp., Mainz, Germany) plates were used as the host substrates. The fs laser direct writing procedure was implemented to create 3D-modified patterns in glass. Through a subsequent annealing process followed by wet chemical etching and an additional annealing process, the laser-modified regions can be fully removed to form 3D hollow structures with highly smooth internal walls. Further details of the fabrication procedure can be found in our previous reports^36–38^. The second procedure is 3D electrode patterning of the fabricated microfluidic structures from the inside out. Optical micrographs and schematics of samples at each step of the preparation process are presented in Figures 1b and c, respectively. After FLAE (the leftmost photo and schematic in Figures 1b and c), water-assisted laser direct-write ablation was carried out using the same fs laser (second image from the left in Figures 1b and c). The laser-ablated region is significantly roughened compared with the non-ablated regions (see the second image from the left in Figure 1b, Supplementary Figures S1 and S2). This allowed us to space-selectively activate the glass surfaces for the metallization of 3D microfluidic structures through the subsequent plating process as described below^36^. The introduction of water to the laser ablation sites enables 3D debris-free microstructuring of glass microchannels^37^. It can be seen that two ~300-μm-wide patterns with a spacing of ~500 μm were generated in region I inside the channel. The pulse energy and writing speed of the fs laser were typically set at 0.25 μJ and 1.5 mm s^−1^, respectively, for the fabrication of microfluidic structures (first procedure). Higher-pulse energies ranging from 1 to 3 μJ were employed to perform in-channel and sidewall ablation of microfluidic structures in the second procedure. For all procedures, a single set of fs laser (FCPA μ-Jewel D-1000, 100 kHz, 457 fs, 1045 nm, IMRA America, Ann Arbor, MI, USA) and an objective lens (M Plan Apo NIR, ×20, Mitutoyo, Kawasaki-shi, Kanagawa, Japan) with a numerical aperture (NA) of 0.40 were used. To perform precise ablation on the internal and sidewall surfaces of a microchannel, the focal spot of the laser beam was first set inside the water-filled channel and at the surface of glass chip, respectively. Next, layer-by-layer scanning with a 5-μm pitch along the *z*-direction was employed until the focusing position reached the internal surface of the channel (scanning was performed across the sidewall surface during sidewall ablation). The subsequent electroless plating process deposits metal thin films only on the laser-exposed regions, thus fabricating 3D electrodes (see red sections in Figure 1a). The metallization process involves electroless copper plating (C-200LT, Kojundo Chemical Laboratory Co. Ltd, Sakado-shi, Saitama, Japan; second from the right in Figures 1b and c) followed by electroless gold plating (K-24N, Kojundo Chemical Laboratory Co. Ltd; the rightmost photo and schematic in Figures 1b and c). The resulting metallic (gold/copper) structures on glass feature strong adhesion to the glass surfaces with good chemical resistance^36^. Before metal plating, the samples were ultrasonically cleaned using a 0.1 mol L^−1^ HCl solution. To ensure rapid deposition of the initial metal layer, a 0.5–1 h static copper plating run was first performed at 50–55 °C. Then, stirring copper plating was carried out at the same temperatures for 1–10 h after replacing the solution with a new solution. Finally, stirring gold plating was performed at 85–90 °C for 1–12 h. The two-step electroless plating process enables high selectivity in metal deposition^36,37,39,40^. Figure 1d shows magnified images of regions I–IV in Figure 1b (from left to right). A comparison of I and II (the same region before and after plating) reveals clearer evidence for the selective deposition of copper thin films on the laser-ablated regions. In regions III and IV, one can see the formation of vertical electrodes with a height of ~610 μm on the sidewalls. Although the formed metal films were somewhat roughened (Supplementary Figure S3), the roughness of the microelectric pads did not significantly influence the electrical manipulation of microorganisms. To improve the roughness of the electric pads, thermal or laser annealing and the further optimization of the metal plating process might be effective. In principle, our technique can easily prepare electrodes with an aspect ratio (height/thickness) >60 at any position on the sidewalls of closed glass microchannels. The prepared samples were cleaned and dried for electrical interconnection to the external power supply. Copper wires were bonded to the electrodes prepared on glass surfaces using a silver paste (EPO-TEK H20E, Epoxy Technology, Inc., Billerica, MA, USA). To solidify the silver paste, the bonded samples were heated at 90 °C for 1 h or at 120 °C for 30 min. Electrical isolation of the fabricated metal microstructures in the liquids that would be introduced into the microchannel during the electro-orientation of microorganisms was achieved by dip-coating with a mixture of SU8 solution (SU8-2050, Microchem Corp., Newton, MA, USA) and acetone (volume ratio=1:30) followed by baking at 90 °C for 30 min.

### Electro-orientation of microorganism motions in fabricated electrofluidic devices

*Euglena gracilis*, an aquatic unicellular microorganism, was used as a biological sample for electro-orientation in the fabricated electrofluidics. *Euglena gracilis* strain Z (NIES-47) was obtained from the Microbial Culture Collection at the National Institute for Environmental Science (NIES), Tsukuba, Japan and cultured at 23 °C in an HUT medium (NIES). Illumination control was carried out to obtain 12-h brightness and 12-h darkness in a day. The concentration of cells was maintained at ~10^5^ to 10^6^ mL^−1^. Before manipulation, the conductivities of the liquid medium for *Euglena* cells were measured with a compact conductivity meter (B-771, Horiba Scientific, Kyoto, Japan). Then, the medium was introduced into the fabricated electrofluidic devices using a pipette. The devices were connected to a dual-channel multifunction generator (WF1974, highest frequency: 30 MHz, maximum output voltage: 20 Vp-p/open; NF Corp., Yokohama, Japan). The behavior of the manipulated cells was observed by an optical microscopy system (BX51, Olympus Inc., Tokyo, Japan) and a CCD camera (DP26, Olympus Inc.) at room temperature.

### Dynamic observation of flagellar motions of microorganisms

Observation of flagellar motions of *Euglena* cells in electrofluidic devices was performed under a differential interference contrast (DIC) microscopy system (IX71, Olympus Inc.) equipped with a CCD camera (FASTCAM SA3 Model 120K-C3, 500–1000 fps, shutter speed: 1 ms; Photron Limited, Tokyo, Japan). Two water-immersion objective lenses (Olympus Inc., ×60, NA 0.9) were used for imaging and illumination. To further magnify the images, eyepieces with ×2 and ×5 lens were used. Red light filtered from the lamp source was used for illumination because it has less of an effect on *Euglena* cells than green and blue light. The focus was set at the middle plane of the microchannel along the *z*-direction. The acquired data, including images and videos, were recorded by the Photron Fastcam Viewer (Photron Limited, www.photron.co.jp) and analyzed using ImageJ software^41,42^ (NIH, Bethesda, MD, USA, https://imagej.nih.gov/ij/).

## Results and discussion

### 1D and 2D electro-orientation of microorganisms in closed glass microfluidics

**Figure 2:**
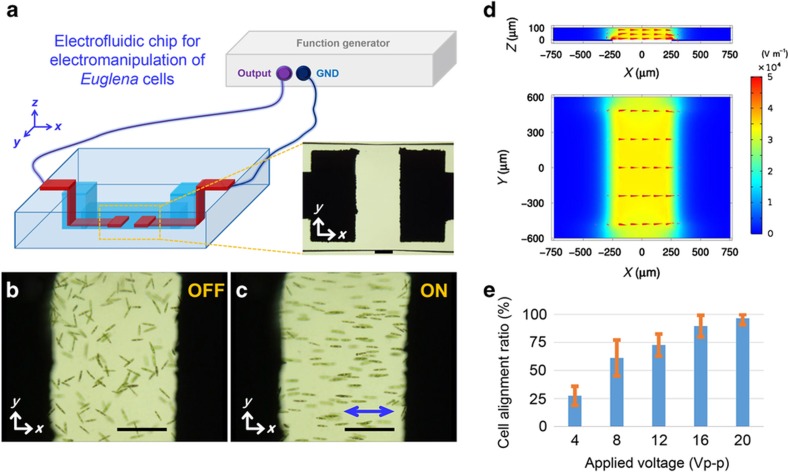
(**a**) 3D schematic of an electrofluidic device consisting of a closed microchannel integrated with a pair of planar microelectrodes for 1D electromanipulation of *Euglena* cells. The inset shows an optical micrograph of microelectrodes in the microchannel. Swimming behavior of *Euglena* cells between a pair of microelectrodes in the (**b**) absence and (**c**) presence of an electric field (20 Vp-p, 0.5 MHz). ‘ON’ and ‘OFF’ correspond to experiments with and without electric power, respectively. The blue arrow indicates the electric field direction, which is the same as the swimming direction of the oriented cells. The scale bars indicate 200 μm. (**d**) Simulated electric-field distributions in the central areas of the *X*–*Z* and *X*–*Y* planes in the microchannel in (**a**–**c**). The simulation parameters are as follows: the electrical conductivities of the metal electrode and the *Euglena* cell solution are 2.17×10^7^ S m^−1^ (Ref. 36) and 0.096 S m^−1^, respectively. The refractive indices of the solution and the glass are 1.333 and 1.515, respectively. The electric potential difference between the electrodes is set to 20 V. (**e**) Quantitative analysis of the electro-orientation of cell motions in the electrofluidics, showing the cell alignment ratio versus applied voltage (Vp-p). The frequency of the applied electric field and the conductivity of the *Euglena* cell solution are maintained at 0.5 MHz and ~0.091 S m^−1^, respectively.

To demonstrate the flexible manipulation of microorganism motions in glass microfluidics, we prepared two types of electrofluidic devices using our hybrid fs laser microfabrication technique. Figure 2a shows a 3D schematic of the electrofluidic device for 1D manipulation of microorganisms in microfluidics. Two electrically isolated electrodes, both ~1×0.5×0.01 mm in size (see the inset of Figure 2a), were fabricated opposite each other with a spacing of 0.5 mm on the bottom surface of a closed straight microchannel with a height of 0.1 mm. Figures 2b and c show the swimming behavior of *Euglena* cells when the alternating current (AC) electric field (20 Vp-p, 0.5 MHz) was turned off and on, respectively. The random swimming behavior of the cells (see Supplementary Video S1) switched to bidirectional swimming, aligned with the electric field direction (see simulated electric field in Figure 2d), as soon as an AC electric field was applied between the two electrodes because of the generation of orientational torques acting on the cells through interactions between the dipole moments induced in the elongated cells by the applied electric field and the field itself^23^. When the body axis of the elongated cell is aligned with the field, the orientational torque exerted on the cell is zero. It should be noted that the electric field does not induce the movement of either the cells or fluids but instead can align the cells. Therefore, the observed phenomenon is attributed to electro-orientation, not to either dielectrophoresis or electro-osmosis. In other words, the motions of the *Euglena* cells are primarily driven by their flagella, other than the applied electric field. Such 1D manipulation is reversible and reproducible with this device (Supplementary Video S2), which is consistent with our previous results^36^. To evaluate the orientation rate of *Euglena* cells using electrofluidic devices, we adopted a cell alignment ratio, defined as the ratio of the number of aligned cells (*N*_A_) to the number of total cells (*N*_T_). In our experiments, we identified oriented cells using two criteria: (i) the direction of motion of the aligned (oriented) cells should be parallel or nearly parallel to the electric field direction (body angle with respect to the electric field direction⩽10° (Ref. 43)); and (ii) oriented cells should always maintain the direction of motion between the electrodes. To quantitatively analyze the efficiency of the electro-orientation of cell motions, we used ~150–160 cells for each experiment. In each experiment, a different voltage was applied (Supplementary Table S1) while the other experimental conditions remained unchanged (the AC frequency and conductivity of the cell solution were maintained at 0.5 MHz and ~0.091 S m^−1^, respectively). The relationship between the cell alignment ratio (*N*_A_/*N*_T_) and the applied voltage is plotted in Figure 2e. When a voltage of 4 Vp-p was applied between two electrodes separated by 500 μm, ~27.5% of the cells were aligned along the electric field. With increasing applied voltage, the cell alignment ratio increased because of the enhancement of the electrical torques exerted on the cells. When voltages of 16 and 20 Vp-p were applied, ~89.6 and ~96.6% of the cells could be oriented, respectively, demonstrating high-efficiency orientation capability. Importantly, no cell damage was observed visually, even at these voltages. In the absence of an electric field, we found that only ~18.8% of the cells temporarily swam along the longitudinal direction of the microchannel (corresponding to the electric field direction when the electric field was applied). However, they randomly changed their direction of motion in 3D without maintaining their swimming direction. In addition, the cells’ swimming speed (10–50 μm s^−1^) in the experiments did not exhibit significant changes (Supplementary Figures S4 and S5) for different applied voltages (Vp-p: 0–20 V). We also investigated the dependence of cell alignment on AC frequency. In the moderate frequency range from 1 kHz to 20 MHz, most of the *Euglena* cells (alignment ratio over 80%) can be oriented along the electric field with a voltage of 20 V. Specifically, the alignment ratios at 1, 5, 10, and 20 MHz were evaluated as 88.4, 83.5, 88.1, and 81.6%, respectively. Under these conditions, no obvious cell damage was observed even after 1 h of the experiment. On one hand, when the AC frequency was reduced to 0.5  and 0.1 kHz under the same voltage, some *Euglena* cells lost their motility and adhered on the channel walls probably due to thermal damage caused by the Joule heating effect. On the other hand, a higher frequency of 30 MHz (the upper frequency limit of the function generator used) reduced the alignment ratio to ~59.9%, indicating the frequency-dependent nature of the electric-field-induced torque aligning the cells. Thus, in most of our electro-orientation experiments, for efficient in-channel alignment of the *Euglena* cells along the electric field, a frequency between 0.5 and 2 MHz was chosen. In addition, we have not observed the orientation perpendicular to the direction of the electric field for the current experimental conditions, which can be ascribed to the high conductivity of the suspension (0.091–0.096 S m^−1^) used in our experiments compared with the previous research (e.g., ~0.001 S m^−1^ (Refs. 43,44,45)) because no perpendicular orientation has been observed for a suspension conductivity beyond 0.05 S m^−1^ in Ref. 23.

**Figure 3:**
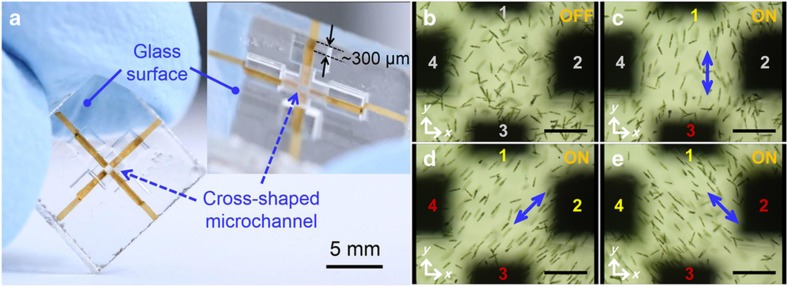
(**a**) Photograph of the electrofluidic device in which two pairs of opposing microelectrodes are formed on the bottom surface of a cross-shaped closed glass microchannel for 2D electromanipulation of *Euglena* cells. The inset is a tilted view of the same chip, showing the preparation of continuous metal structures from inside of the microchannel to the glass chip surface. The depth of the channel bottom from the glass surface is ~300 μm. Swimming behaviors of *Euglena* cells in a microfluidic environment in the absence of an electric field (**b**) and under an applied electric field (20 Vp-p, 0.8 MHz) between electrodes ‘1 and 3’ (**c**) and ‘1 and 3’ and ‘2 and 4’ (**d** and **e**). (**c**), (**d**), and (**e**) represent, respectively, 90°, +45°, and −45° orientations of cell motion with respect to the *x*-axis. ‘ON’ and ‘OFF’ correspond to experiments with and without electric power, respectively. The numbers in gray in (**b** and **c**) indicate that no AC voltage was applied. The numbers in yellow and red in (**c**–**e**) represent the opposite polarities. The blue arrows in (**c**–**e**) indicate the directions of the electric field. The scale bars in (**b**–**e**) indicate 200 μm.

**Figure 4:**
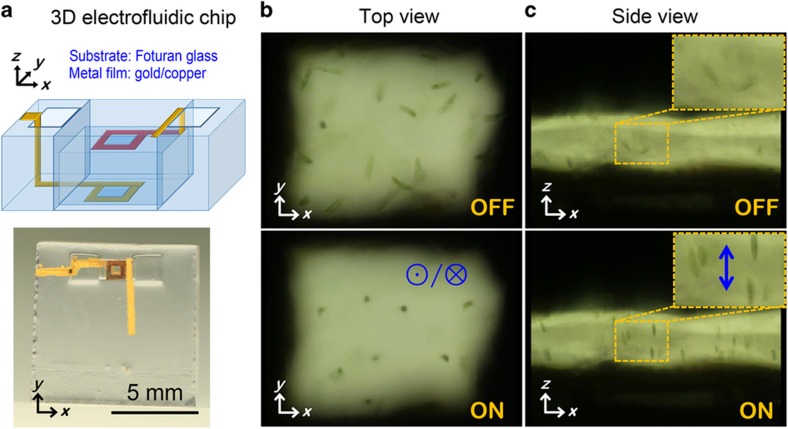
(**a**) 3D schematic and photo of an electrofluidic chip for *z*-directional manipulation; (**b**) top and (**c**) side views of *Euglena* cell motion in a microfluidic channel. Black areas in (**b** and **c**) correspond to the electrodes. ‘ON’ and ‘OFF’ indicate with and without electric power (20 Vp-p, 2 MHz), respectively. The insets in (**c**) show close-up side views of the transparent window area in the microchannel. The symbol ‘⊙/⊗’ in (**b**) and the blue arrow in (**c**) indicate the direction of the generated electric field, which induces bidirectional swimming of cells. The side length of the observation window in (**b**) and the electrode spacing in (**c**) are both ~500 μm.

In contrast to conventional fabrication techniques, one of the distinct features of the proposed technique is the capability for selective metallization of the microchannel walls, which plays a vital role in electrical interconnection between internal electrodes and external metal pads in the fabricated electrofluidic devices for 1D, 2D and even *z*-directional manipulation (Figures 1d, 2a, 3a, and 4a). It should be noted that this capability also enables us to form two vertical electrodes on the channel walls such that the two electrodes are positioned face to face. Such a configuration allows us to produce a completely uniform electric field along the microfluidic channel. A pair of vertical sidewall electrodes has been successfully applied to flexibly control the movement of *C. elegans* in channels based on electrotaxis in our previous study^37^. In this study, however, two electrodes were formed on the bottom of the microchannel, which remained efficient for performing the in-channel alignment of *Euglena* cells in 1D, as demonstrated. One of the reasons for using this configuration is that the height of the channel in this work is only 0.1 mm, so the influence of the nonuniformity of the electric field on the electrical alignment of *Euglena* cells within such a height could be neglected under our experimental conditions when a voltage of 20 V was applied. The simulation shown in Figure 2d confirmed that although the largest electric field strength was produced at peripheral regions of the edges of the electrodes on the channel bottom, the electric field intensities between the channel top (*Z*=100 μm) and the channel bottom (*Z*=0) in the middle area (from *X*=−200 μm to *X*=200 μm) were on the order of ~3×10^4^ V m^−1^. Indeed, using this configuration, a voltage of higher than ~16 V, which corresponds to an electric field intensity on the order of ~2×10^4^ V m^−1^, is high enough to align most of the cells in the channel along the channel wall, as described in Figure 2e. Another reason for using this configuration is to shorten the distance between the two electrodes to minimize the applied electric voltage.

Furthermore, 2D manipulation of cell motion, enabling flexible control of the alignment direction in microfluidic channels, can be performed by arranging the positions and configurations of the electrodes. Figure 3a shows another electrofluidic device in which two pairs of opposing microelectrodes are formed on the bottom surface of a cross-shaped glass microchannel for 2D manipulation. When the *Euglena* cells were introduced into the device, random swimming behavior is observed (Figure 3b). By applying an AC electric field between appropriate combinations of electrodes, 2D orientation of cell motions can be achieved in the microfluidic environment. Specifically, when an electric field (20 Vp-p, 0.8 MHz) was applied between electrodes 1 and 3, as shown in Figure 3c, the swimming of most cells in the central area of the chip was aligned along the *y-*direction (perpendicular to the horizontal axis: 90° orientation). A more complex orientation can be achieved by applying electric fields between different pairs of electrodes. For example, when electric fields were applied simultaneously between two pairs of electrodes (‘1 and 3’, and ‘2 and 4’), in which electrodes labeled with numbers of the same color have the same polarity while those labeled with numbers of different colors have the opposite polarity, the +45° and −45° orientation of cell motions with respect to the *x*-axis can be demonstrated, as shown in Figures 3d and e, respectively. Moreover, temporal control of the electric field patterns by switching pairs and polarities of electrodes enables dynamic control of cell motion in the microchannels (e.g., see Supplementary Video S3).

### *Z*-directional electro-orientation of microorganisms in glass microfluidics

**Figure 5:**
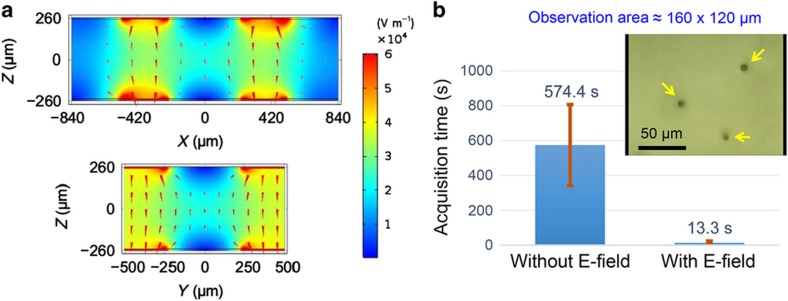
(**a**) Simulated electric field distributions at the center of the *X*–*Z* and *Y*–*Z* planes generated by the electrodes with square outlines on the top and bottom surfaces of a microfluidic channel. The simulation parameters are as follows. The width and thickness of the electrodes are 250 and 10 μm, respectively. The center of the electrode has a transparent window with an area of 500×500 μm. The electrical conductivity of the metal electrode and the *Euglena* cell solution are 2.17×10^7^ S m^−1^ (Ref. 36) and 0.096 S m^−1^, respectively. The refractive indices of the solution and the glass window are 1.333 and 1.515, respectively. The electric potential difference between the top and bottom electrodes is set to 20 V. (**b**) Comparison of the average acquisition time required for capturing images of five *Euglena* cells swimming continuously along the *z*-direction in a microfluidic channel with and without an electric field (20 Vp-p, 0.8 MHz). The inset shows three manipulated cells (see arrows) in an imaging area of ~160×120 μm. Observations were repeated 10 times for each scheme. The conductivity of the *Euglena* cell solution is ~0.096 S m^−1^.

To minimize the side effect on biological samples of light illumination and thereby to perform experiments on dynamic observation of microorganism motions under natural conditions, it is important to reduce the data acquisition time (for images, videos, and so on). In addition, with elapsed time, the conditions of microorganism suspensions may change, for example, because of an increase in the suspension temperature as a result of exposure to the microscope light, especially when a high-NA condenser (objective lens) is used. Thus, collecting enough data in a limited amount of time requires improvement in the efficiency of observing microorganism motions along a specific direction. *Z*-directional (perpendicular to the top and bottom surfaces of the microfluidic channel) electromanipulation is expected to improve the efficiency of front-view observation of continuously swimming microorganisms^17,19,32,46^. To this end, electrodes with a square outline (side length of outer square ≈1000 μm, side length of inner square (observation window) ≈500 μm, thus electrode width ≈250 μm), shown in Figure 4a, were prepared on the top and bottom of the interior walls of a microchannel (~1100 μm wide, ~500 μm high) using hybrid fs laser microfabrication. The open square at the center of the electrode is necessary to observe *z*-directionally oriented cells from the top glass surface, enabling front-view observation. To connect the electrodes with an external function generator, 3D metal wiring was formed by the selective metallization of sidewalls and glass surfaces using the same technique (Figure 4a). As one can imagine, it is not easy to construct such a 3D metal wiring structure in 3D microchannels, and many complicated procedures are required even when using existing techniques. In contrast, this technique can create the structure with little difficulty. As seen in the upper photos in Figure 4b (top view) and Figure 4c (side view), the *Euglena* cells in the microchannel were swimming randomly when no electric field was applied. Once an electric field (20 Vp-p, 2 MHz) was applied between the two electrodes, the motion of the *Euglena* cells changed markedly, becoming oriented bidirectionally along the electric field direction. In particular, the side view of the lower photo in Figure 4c confirmed that the swimming of *Euglena* cells was oriented along the *z*-direction. Most of the cells were *z*-directionally oriented under the applied electric field (see the inset of the lower photo in Figure 4c). Although the transparent window area at the center of the electrodes was not coated with metal, the surrounding electrode patterns generated sufficiently high electric field intensities for *z*-directional orientation. To confirm this, the electric field distribution inside the microfluidic channel was calculated through numerical simulation, as shown in Figure 5a. We can see that an electric field with an intensity on the order of 2×10^4^ V m^−1^ can be generated in the transparent window area, and the largest electric field strength in the window area is obtained in the middle plane of the channel along the *z*-direction (*X*–*Y* plane at *Z*=0). Moreover, the arrow plots in the middle *X*–*Y* plane of the window area indicate that the generated electric field is distributed predominantly along the *z*-direction. This is because most of the *x*- and *y*-direction components in that area are cancelled out because of the spatial configuration of the electrodes, although the top and bottom electrode structures, including the external metal interconnection pads, are not strictly symmetrical. Clearly, this vertical electric field plays a major role in *z*-oriented electromanipulation.

To test the observation efficiency of the swimming of *z*-directionally oriented cells using the fabricated electrofluidics, a high-density cell solution (Supplementary Video S4) was used. The number of oriented cells was counted in the window area of the electrodes using an imaging area of ~160×120 μm in the middle *X*–*Y* plane of the microchannel. As many as 45 *Euglena* cells were successfully observed to swim continuously along the *z*-direction within 1 min when an electric field (20 Vp-p, 0.8 MHz) was applied between the electrodes (Supplementary Video S5), thus demonstrating the device’s high-efficiency capability for front-view observation. As shown in the inset of Figure 5b, by narrowing the imaging area, the device showed the capability for capturing an image of three oriented cells at a time, even though the acquisition rate of the swimming cells decreased. Next, we compared the average times required to capture the images of five *Euglena* cells swimming along the *z*-direction in the same area with and without an electric field (20 Vp-p, 0.8 MHz). In both cases, the same cell solution (Supplementary Video S4) was used and the experiment was repeated 10 times (Supplementary Table S2). In the absence of the electric field, the average time was 574.4 s. Remarkably, this time was reduced to 13.3 s under the electric field. It is therefore concluded that the average time required for continuous observation of five *Euglena* cell swimming along the *z*-direction was shortened by a factor of ~43 (Figure 5b) with our device. In addition to *z*-directional electromanipulation of cells, more flexible, high-efficiency 3D manipulation of cell motions for observation from various directions is expected to be made possible through the further development of novel electrofluidics.

### Bidirectional swimming of *Euglena* cells in response to AC electric fields

**Figure 6:**
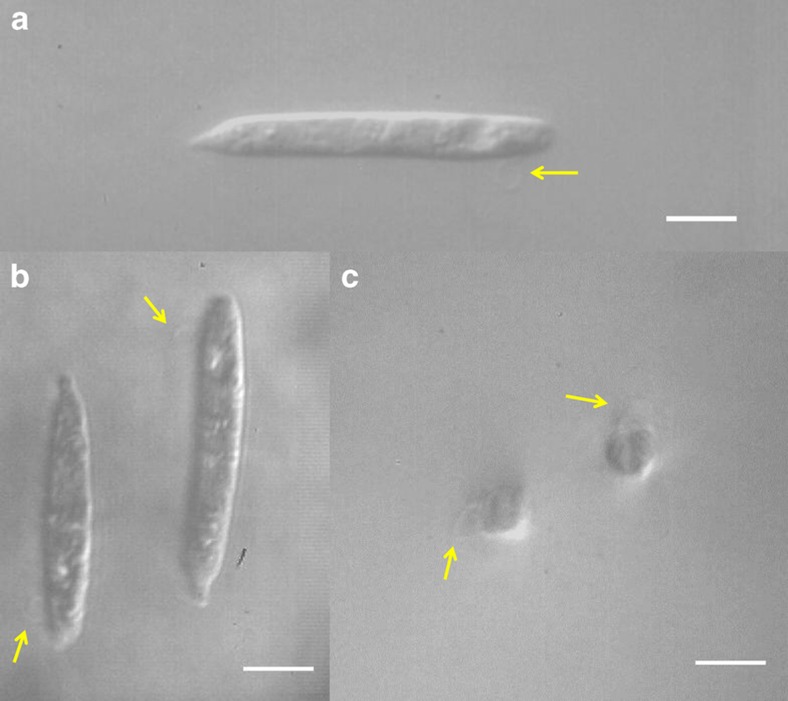
*Euglena* cells electro-oriented along the (**a**) *x*-direction, (**b**) *y*-direction, and (**c**) *z*-direction in the fabricated electrofluidic devices. The arrows indicate the positions of the flagella. The scale bar indicates 10 μm.

Combining the fabricated electrofluidic devices and a DIC microscope with a high-speed camera enables us to perform a controllable and in-depth observation of the motions of specific parts of the cells manipulated by the applied AC electric field. Specifically, flagellar motions of the cells can be observed from the various angles. Figure 6 shows snapshots of the flagella when observing the *Euglena* cells electro-oriented along the (a) *x*-, (b) *y*-, and (c) *z*-directions.

**Figure 7:**
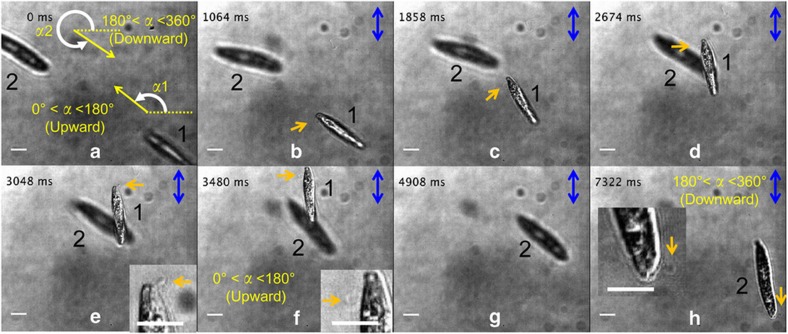
Time-sequential images of bidirectionally swimming behaviors of two *Euglena* cells (cell 1 swims upward and cell 2 swims downward) before and after applying an electric field. (**a**) Image taken just before applying the electric field. Dashed lines indicate the horizon direction, which is perpendicular to the direction of the applied electric field. Yellow and white arrows indicate the cells’ initial body axes and body angles, respectively. The estimated body angles of cell 1 and cell 2 are ~142.7° and ~326.6°, respectively. (**b**–**h**) Images taken just after applying the electric field. The inset in (**h**) shows the close-view image of the flagellum of cell 2, which swims downward, suggesting that its initial body angle is larger than 180°. The brown arrows indicate the positions of the flagella. The blue arrows show the direction of the electric field. The scale bar indicates 10 μm.

Although it is well known that the swimming of *Euglena* cells is primarily driven by the beating of their flagella, sequential photographs of their movements can identify the positions of flagella at specific times, thus offering the possibility of determining the dominant factors on the swimming direction of cells and knowing the driving force of their swimming. Such observation can reveal why the AC electric field application elicits bidirectional swimming of *Euglena* cells^36^. Here we define the body angle *α* as the angle of the body axis of the cell (the side where a flagellum is equipped is the top) with respect to the horizontal line perpendicular to the direction of the applied electric field (Supplementary Figure S6). Therefore, to determine the body angle, it is essential to identify the position of the flagellum. The sequential images shown in Figure 7 revealed that the initial body angles before applying the electric field determined the direction of subsequent swimming oriented by the field. In most cases, *Euglena* cells swim along the direction with an angle of ~90° (cell 1 swimming upward in Figure 7f) when the initial body angle is between 0° and 180° (for cell 1, *α*1≈142.7°), whereas the angle is ~270° (cell 2 swimming downward in Figure 7h) when the initial body angle is between 180° and 360° (for cell 2, *α*1≈326.6°). We confirmed this fact by repeating the observation many times. In the cases of nearly 0° and 180°, the final swimming directions depend on the next moment of the body angle because the swimming direction of *Euglena* cells dynamically varies with time. Because the combination of dynamic imaging and functional microfluidic devices has provided a useful and efficient approach to investigate the locomotion mechanisms in many biological processes^47–50^, we believe that in the near future, the controllable alignment of cell swimming in a 3D microspace of microfluidics will facilitate the detailed analysis of the flagellar motion. In particular, the *z*-directional cell alignment shown in Figure 6c enables the observation from the front side of the cells, which will be very useful for the 3D analysis of flagellar motions. In addition, the electrofluidic devices can align multiple cells in the same direction for parallel observation, which is expected to further increase the acquisition rate of experimental data, reproducibility, and accuracy in measurements.

## Conclusion

New electrofluidic devices based on monolithic integration of metallic microelectrodes into 3D microchannels in glass were prepared using a newly developed hybrid subtractive and additive fs laser microfabrication technique. The electrofluidic devices fabricated using this technique were applied to nanoaquariums for the efficient observation of aquatic microorganisms. The integration of one pair and two pairs of opposing planar electrodes successfully demonstrated 1D and 2D flexible manipulation of *Euglena* cells in microfluidic channels. Furthermore, the fabrication of electrodes with square outlines at the top and bottom of the interior walls of a microchannel enabled the observation of cells swimming parallel to the direction of the body motion (front-view observation) with an efficiency enhanced by a factor of ~43 relative to the case of no electromanipulation. The fabricated electrofluidic devices successfully demonstrate observation of specific parts of the cells, such as the flagella, under the motion control in 3D. In addition to *Euglena* cells, the fabricated electrofluidic devices can be applied to the 3D electrical alignment of other elongated cells and microorganisms. Because most of the observation can be performed through the transparent glass area and high-NA objective lenses can be used for imaging and illumination in fabricated electrofluidic devices, we believe that these devices can also be used for the high-resolution fluorescence imaging of labeled biological samples. This paper primarily described a single-step procedure of laser ablation for the space-selective modification of glass microchannels followed by electroless plating for the selective metallization of 3D microfluidic devices. In principle, the post-fabrication of microelectric patterns on prefabricated metal pads for the preparation of second overlapped pads remains possible, although the interference of prefabricated metallic films on post-modified regions during the plating process can occur. To overcome this problem, the composition of the electroless plating solution must be carefully optimized to avoid any reaction with the prefabricated pads. Compared with conventional electrode fabrication methods, this technique provides a simpler scheme to reliably install electrodes with flexible configurations at any position in 3D microfluidic structures. In addition to the development of the 3D continuous control of the electro-orientation of cells and particles, we expect that this technique can be extended to the fabrication of different types of electrofluidic devices for 3D dielectrophoretic manipulation of biological samples^51–53^, 3D electrorotation in microscale spaces^54,55^, and electrical impedance sensors^56,57^. The incorporation of photonic components, such as optical waveguides, fibers, and microlenses^38^, into the fabricated electrofluidic devices using the same fs laser would significantly enhance the performance of biochips, paving the way to 3D ‘all-in-one’ lab-on-a-chip devices.
