# Supplementary material for: Controllable alignment of elongated microorganisms in 3D microspace using electrofluidic devices manufactured by hybrid femtosecond laser microfabrication
Source: Microsyst Nanoeng. 2017 Feb 27;3:16078. doi: 10.1038/micronano.2016.78 (PMC6444996; doi:10.1038/micronano.2016.78)
Supplement: Supplementary Information [file micronano201678-s1.pdf]

Supplementary file

Controllable alignment of elongated microorganisms in a 3D microspace using electrofluidic devices manufactured by hybrid femtosecond laser microfabrication

Jian Xu[1], Hiroyuki Kawano[2], Weiwei Liu[3], Yasutaka Hanada[1], Peixiang Lu[3,4], Atsushi Miyawaki[2], Katsumi Midorikawa[1] and Koji Sugioka[1]

Microsystems & Nanoengineering (2017) **3,** 16078; doi:10.1038/micronano.2016.78; Published online: 27 February 2017

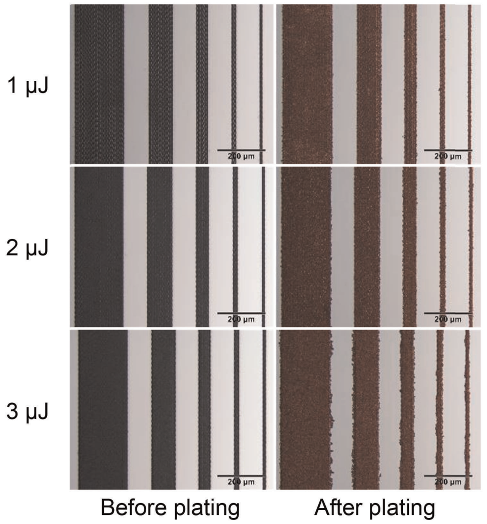

Figure S1 Selective metallization of Foturan glass surfaces using femtosecond laser direct-write ablation followed by electroless metal plating. Optical microscope images of femtosecond laser ablated pads with different linewidths under different pulse energies (1, 2, and 3 μJ) before electroless plating (dark area in the left panel) and after electroless plating of copper (red area in the right panel).

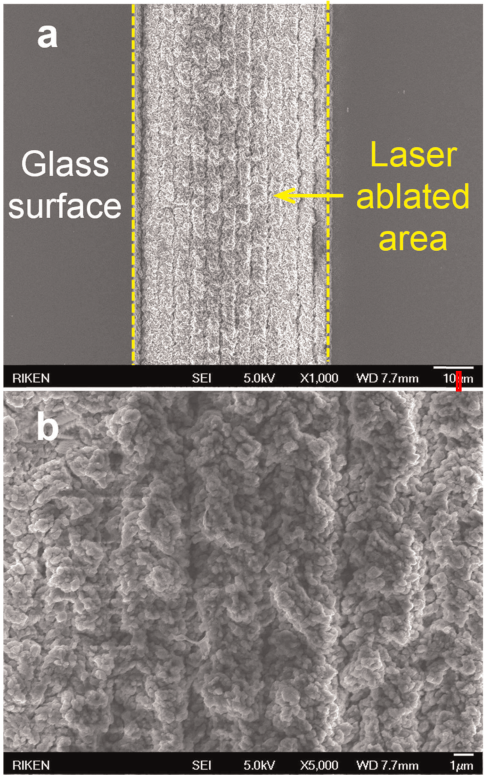

Figure S2 SEM images of laser ablated area on the Foturan glass surface at (**a**) low magnification and (**b**) high magnification. In selective metallization process, generation of sufficient roughness by the laser ablation allows the copper atoms precipitated in the solution to be adhesively captured. Roughened surface can be clearly observed in Figure S2. Aggregated-particle like structures can be found in Figure S2b.

CAPTIONS FOR SUPPORTING VIDEOS

Video S1. *Euglena* cells swim randomly between a pair of electrodes in a microfluidic channel when no electric field was applied. Black areas on the left and right correspond to electrodes.

Video S2. Temporal (OFF-ON-OFF) control of electro-orientation of *Euglena* cells. At the beginning (0 s), *Euglena* cells are swimming randomly since no electric field was applied. As soon as the electric field (20 Vp-p, 0.5 MHz) is applied (at ~1 s), the random swimming of *Euglena* cells switches to bidirectional swimming. When the electric field is removed (at ~11 s), they return to random swimming. Black areas on the left and right correspond to electrodes.

Video S3. Dynamic control of the electro-orientation direction of *Euglena* cells in a microfluidic environment with temporal control of electric field directions by changing the combinations of electrodes applied with voltages (20 Vp-p, 0.8 MHz). At the beginning (0 s), the angle of the electric field direction (alignment angle of oriented *Euglena* cells) is 45° with respect to the

[1]RIKEN Center for Advanced Photonics, 2-1 Hirosawa, Wako, Saitama 351-0198, Japan; [2]Laboratory for Cell Function Dynamics, RIKEN Brain Science Institute, 2-1 Hirosawa, Wako, Saitama 351-0198, Japan; [3]Wuhan National Laboratory for Optoelectronics and School of Physics, Huazhong University of Science and Technology, Wuhan 430074, China and [4]Laboratory of Optical Information and Technology, School of Science, Wuhan Institute of Technology, Wuhan 430073, China.
Correspondence: Jian Xu (jxu@riken.jp) or Koji Sugioka (ksugioka@riken.jp)

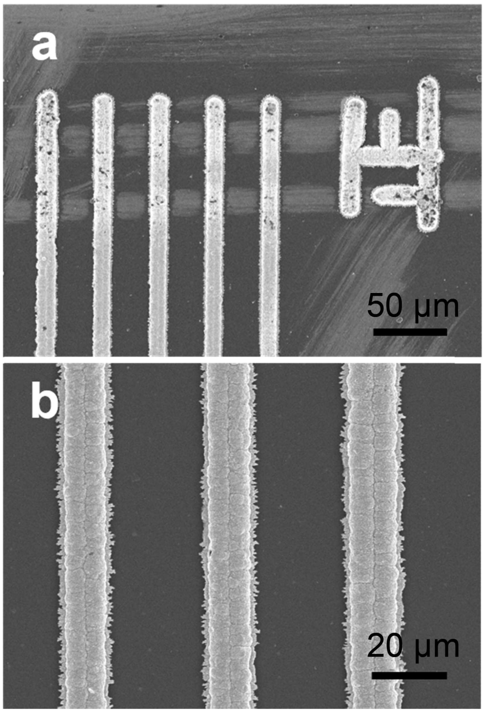

Figure S3 SEM images of fabricated metallic lines on the Foturan glass surface at (**a**) low magnification and (**b**) high magnification. To fabricate these lines, metallization of laser ablated regions were performed by two-step electroless plating.

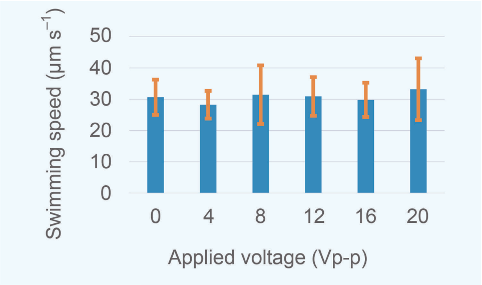

Figure S4 Swimming speed of the cells versus applied voltage (I). The speed was estimated from the translation distance in 5 s at each voltage (0, 4, 8, 12, 16, 18, 20 Vp-p). For each condition, the data of ten cells was collected. **Note:** Swimming speed of the *Euglena* cells depends on many factors such as types of strain, sizes and living conditions of the cells, the viscosity, composition and temperature of cell suspension, and light illumination[1,2]. In the experiments for evaluating the effects of electric field intensity, swimming speed of the *Euglena* cells was estimated at 10 ~ 50 µm s^{-1}, which does not exhibit significant changes regardless of presence of electric field as well as the intensity of electric field (Vp-p: 0, 4, 8, 12, 16, 20 V, electrode spacing: 500 µm, frequency: 0.5 MHz, conductivity: 0.091 S m^{-1}).

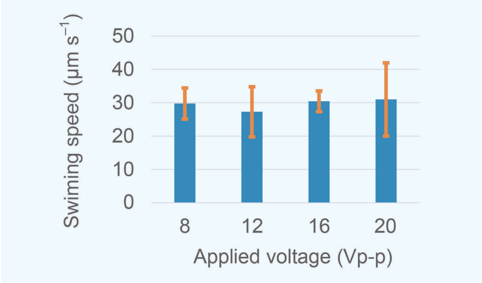

Figure S5 Swimming speed of the cells versus applied voltage (II). The speed was estimated from the omnidirectional swimming time of the oriented cells in a 500 µm distance at each voltage (8, 12, 16, 20 Vp-p). For each condition, the data of ten cells was collected. **Note:** At the voltages of 0 and 4 Vp-p, few cells could swim along the same direction for 500 µm.

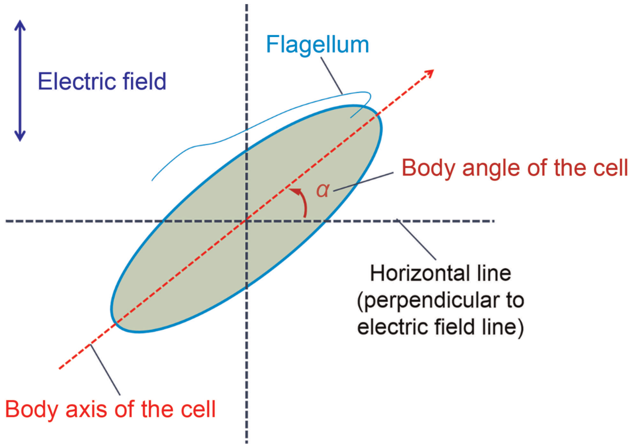

Figure S6 Schematic of the body angle (α) of the cell, which is defined as the angle of the body axis of the cell (dashed red line, the side where a flagellum is equipped is top) with respect to the horizontal line perpendicular to the direction of the electric field. The direction of the red arrow indicates the flagellar position of the cell. The blue arrow indicate the direction of the electric field.

horizontal axis. Then (at ~ 5 s), the angle changes to 0° (the horizontal axis). Finally (at ~ 13 s), the angle changes to − 45° relative to the horizontal axis.

Video S4. *Euglena* cells swim randomly in the window area on the middle *X-Y* plane of a microfluidic channel integrated with a pair of electrodes with square outlines on the top and bottom of interior walls of a microchannel when no electric field was applied. The imaging area was about 160 × 120 µm.

Video S5. *Z*-directional control of *Euglena* cell motions when an electric field (20 Vp-p, 0.8 MHz) was applied between the top and bottom electrodes with square outlines in the microfluidic environment. The imaging area (ca. 160 × 120 µm) was located on the middle *X-Y* plane of the microfluidic channel. In 1 min, about 45 cells oriented along the *z*-direction to swim were successfully observed.

Table S1 Alignment ratio (N_A/N_T) at different applied voltages

Voltage (Vp-p)	Alignment ratio (N_A/N_T) of individual frames							Mean	STD dev
0	7/34	8/38	6/29	8/35	9/30	7/39	7/36	0.188	0.061
	5/40	10/45	5/47	6/46	4/45	10/40			
4	5/17	2/16	4/15	3/10	3/14	3/12	7/23	0.275	0.085
	3/15	7/17	8/21						
8	5/8	9/11	17/23	10/15	10/13	11/18	10/15	0.611	0.161
	12/18	6/13	4/10	3/10					
12	4/6	9/10	9/10	8/11	9/12	7/11	6/8	0.727	0.098
	9/13	7/10	8/10	6/9	6/11	8/12	10/12		
	6/9								
16	13/14	13/16	12/14	16/19	16/16	13/14	16/18	0.896	0.097
	18/18	7/10	16/16						
20	7/7	7/7	6/6	5/5	6/6	7/7	8/8	0.966	0.059
	7/7	9/11	13/15	11/11	10/11	13/13	9/10		
	12/13	9/9	11/11						

Each frame was selected from the recorded videos with an interval of ~10 s. The number of frames selected from the recorded videos was over 10 for each applied voltage.

Table S2 Acquisition time for the observation of five *Euglena* cells swimming continuously along the *z*-direction in a microfluidic channel with and without an electric field (20 Vp-p, 0.8 MHz)

Measured time	Acquisition time (s)	
	Without E-field	With E-field
01	376	22
02	410	14
03	351	12
04	615	5
05	257	9
06	568	38
07	690	9
08	811	6
09	650	13
10	1016	5
Mean	574.4	13.3
STD Dev	233.05	10.09

Medium conductivity: 0.096 S m^{-1}; observation area ≈160 × 120 μm.

REFERENCES

1 Machemer-Röhnisch S, Nagel U, Machemer H. A gravity-induced regulation of swimming speed in *Euglena gracilis*. *Journal of Comparative Physiology A* 1999; **185**: 517–527.

2 Richter PR, Strauch SM, Ntefidou M *et al.* Influence of different light-dark cycles on motility and photosynthesis of *Euglena gracilis* in closed bioreactors. *Astrobiology* 2014; **14**: 848–858.
